# Validity and Test–Retest Reliability of the Spanish Version of the International Hip Outcome Tool (iHOT-12Sv)

**DOI:** 10.3390/jcm11216232

**Published:** 2022-10-22

**Authors:** Ángel González-de-la-Flor, Ibai López-de-Uralde-Villanueva, Juan Antonio Valera-Calero, Jaime Almazán-Polo, José Javier López-Marcos, César Fernández-de-las-Peñas, Pablo García-Fernández, Gustavo Plaza-Manzano

**Affiliations:** 1 Department of Physical Therapy and Sport Medicine, Hospital Universitario Quironsalud Madrid, 28223 Madrid, Spain; 2 Faculty of Sport Sciences, Universidad Europea de Madrid, 28670 Madrid, Spain; 3 Department of Radiology, Rehabilitation and Physiotherapy, Universidad Complutense de Madrid, 28040 Madrid, Spain; 4 IdISSC, Instituto de Investigación Sanitaria del Hospital Clínico San Carlos, 28040 Madrid, Spain; 5 VALTRADOFI Research Group, Department of Physiotherapy, Faculty of Health, Universidad Camilo José Cela, 28692 Villanueva de la Cañada, Spain; 6 Department of Physical Therapy, Occupational Therapy, Rehabilitation and Physical Medicine, Universidad Rey Juan Carlos, 28922 Alcorcón, Spain; 7 Cátedra Institucional en Docencia, Clínica e Investigación en Fisioterapia: Terapia Manual, Punción Seca y Ejercicio Terapéutico, Universidad Rey Juan Carlos, 28922 Alcorcón, Spain

**Keywords:** clinimetrics, function, hip, outcome measures, validity, reliability

## Abstract

**Objective**: To develop a Spanish version of the international Hip Outcome Tool questionnaire (iHOT-12Sv) for assessing the psychometric characteristics (internal consistency, convergent validity, test–retest reliability, and floor and ceiling effects) of this version in physically active patients with hip pain. **Methods**: After conducting the translation and transcultural adaptation, a consecutive sample of patients with labral tear injury and/or femoroacetabular impingement (Pincer or Cam type) were recruited in a Spanish Hospital. Patients completed the iHOT-12Sv and the Spanish version of the iHOT-33 and the Hip Outcome Score (HOS). Internal consistency was calculated using Cronbach’s alpha; convergent validity was evaluated using Spearman correlation coefficients (Rho) with iHOT-33 and HOS; test–retest reliability was examined using the intraclass correlation coefficient (ICC), standard error of measurement (SEM), and minimal detectable changes (MDC); and floor and ceiling effects were calculated as the percentage of patients who obtained the minimum and maximum score. **Results**: One hundred and fifty-three patients (64.7% males) participated in this study. Cronbach’s alpha of 0.92 revealed the excellent internal consistency. In addition, the iHOT-12Sv demonstrated strong to very strong correlations with the HOS (Rho ranged from 0.741 to 0.827; *p* < 0.001) and the iHOT-33 (Rho = 0.932; *p* < 0.001), respectively; acceptable test-retest reliability (ICC = 0.86 to 0.94); SEM = 6.21 and MDC = 17.22; and no floor or ceiling effects were found. **Conclusions**: The iHOT-12Sv can be used as a valid and reliable tool for clinical evaluation of physically active patients with hip pathology. However, the full version is preferable for research purposes aiming to assess changes in hip function.

## 1. Introduction

Young and middle-aged adults with hip pain often present with a confusing overlap of signs and symptoms [1,2]. Recently, three hip-related pain diagnosis classifications were proposed: femoroacetabular impingement (FAI) syndrome; acetabular dysplasia and/or hip instability; and other conditions without distinct osseus morphology (e.g., isolated or combined labral, chondral, and ligamentum teres findings related with tears, cysts, erosions, or hypertrophy) [3].

Because of the abnormal osseus morphology commonly found at the acetabulum/femoral head in FAI, clinicians focus on objective outcomes such as range of motion, strength, and radiographic appearance [4]. However, several radiographic findings suggestive of femoroacetabular impingement are also commonly found in asymptomatic subjects [5], and these outcomes are poor indicators of patients’ function. Considering subjective outcomes of symptoms such as emotional and social health and quality of life is essential to truly assess the patients’ function [4].

Several self-reported questionnaires focused on patients with osteoarthritis or undergoing hip arthroplasty with a limited activity level, which cannot be applicable to young patients with different expectations (e.g., Harris Hip Score, Hip disability and Osteoarthritis Outcome Score, Oxford Hip Score, or Lequesne Index of Severity for Osteoarthritis of the Hip) [6]. Therefore, Mohtadi et al. [4] developed the international Hip Outcome Tool (iHOT-33) as a valid and reliable questionnaire for measuring the physical function and quality of life applicable for young and active patients with hip pathology.

Subsequently, Griffin et al. [7] developed a short version of this iHOT-33 (iHOT-12) based on 12 items from the original version covering four domains: (1) symptoms and functional limitations; (2) sports and leisure time activities; (3) job-related complaints; and (4) social, emotional, and lifestyle complaints [7].

The iHOT-12 was originally developed in English and translated into Turkish [8], Portuguese [9], Dutch [10], French [11], and German [12], but not into Spanish. Although there is a Spanish version of the iHOT-33 [13], the validation of the short Spanish version could be useful for clinicians and researchers for reducing patient burden and administrative effort, given the minimal clinically important difference (MCID) and patient acceptable symptomatic state (PASS) refence values previously reported [14]. Given the high number of Spanish speakers around the world, the aim of this study was to translate and culturally adapt the original iHOT-12 into Spanish to determine its validity and test–retest reliability.

## 2. Materials and Methods

### 2.1. Study Design

An observational study was conducted following the original version of the iHOT-12 study methodology [7] considering the ethical principles of the Declaration of Helsinki. The study protocol was supervised and previously approved by the Clinical Ethics committee of the Hospital Clínico San Carlos (ID: 21/257). The Consensus based Standards for the selection of health status Measurement INstruments (COSMIN) checklist for studies evaluating measurement properties was followed [15]. This study was divided into two stages: (1) translation and transcultural adaptation and (2) analysis of psychometric properties.

### 2.2. Participants

A consecutive sample recruited from the unit of Rehabilitation, Sports Medicine, and Physiotherapy at Hospital Universitario Quirónsalud located in Madrid (Spain) was recruited from April 2021 to February 2022 and consequently screened for potential eligibility.

To be included in this study, patients had (1) to be between 18 and 60 years of age; (2) to be referred for a complaint of hip and/or groin pain for at least 3 months of duration; (3) to provide a medical diagnosis confirmation of labral tear or/and FAI (Cam or Pincer Type); (4) to have sufficient reading and oral comprehension capacity; and (5) to sign the written informed consent. Exclusion criteria were as follows: (1) pregnancy; (2) presence of red flags (i.e., infection, bone fracture, and malignancy processes); (3) previous history of hip lumbopelvic surgery or specific hip diseases (e.g., Legg–Calve–Perthes, avascular necrosis or epiphysioysis); (4) being under medical treatment (e.g., hip anesthetic infiltrations or physiotherapy treatments); and (5) any other metabolic, neurologic, muscular, or cardiovascular condition biasing this study.

Participants were asked to complete and return all questionnaires via mail. Previously, they received an informative letter explaining all of the procedures and the objective of this study. Patients were not involved in the design, conduct, divulgation, or diffusion of our research.

### 2.3. Sample Size Calculation

According to the criteria established by Gorsuch [16] (widely used to evaluate psychometric properties in psychology), a sample size of at least 120 patients (10 per item) would be necessary. Furthermore, considering that, in our study, the factor loadings of the items were >0.60, a sample of at least 4:1 items per factor could be adequate (48 patients) [17]. However, de Winter et al. [18] established the number of 50 observations as the minimum number necessary to perform an exploratory factor analysis (EFA). Therefore, we finally considered it necessary to reach a sample size of at least 120 patients to determine the structure of the Spanish version of the IHOT-12.

### 2.4. Translation and Transcultural Adaptation

Based on recommendations from prior studies [19,20], the questionnaire was initially translated from English into Spanish by two bilingual translators (native Spanish speakers). Both translators, with the research group, performed a synthesis and some minor edits. Retro translation was ensured by involving two native English and Spanish bilingual translators who were blinded to the original version. The two resulting versions were subsequently compared between each other and to the English version of the iHOT-12 [7]. A committee composed of four translators and two researchers revised these versions and prepared a pre-final one. Semantic and conceptual criteria and content equivalences were evaluated. Finally, a pre-test of the pre-final version was conducted by 15 Spanish clinicians to assess the quality of translation, cross-cultural adaptation, and clinical usefulness.

### 2.5. Measuring Instruments

#### 2.5.1. The iHOT-12Sv

The original English iHOT-12 is a valid and reliable disease-specific questionnaire that measures physical function and health-related quality of life in a younger patient population (younger than 60 years) with hip pain conditions [7]. Similarly, the iHOT-12Sv consists of 12 questions that patients answer by placing a mark on a 100 mm visual analogue scale placing greater impairment on the left (0) and absence of problems on the right (100). Final scores were calculated as the average score of all completed questions. Therefore, higher scores reflect better physical function and quality of life in contrast with lower scores. The final version of the iHOT-12Sv is available in Supplementary Materials.

#### 2.5.2. The iHOT-33

The iHOT-33 is a patient-derived and patient-reported outcome instrument designed to measure hip-related quality of life in young adults with non-arthritic hip pain. This questionnaire consists of four domains divided into 33 items: symptoms and functional limitations (16 items); sports and recreational physical activities (6 items); job-related concerns (4 items); and social, emotional, and lifestyle concerns (7 items) [4]. Final scores range from 0 (poorest pain and function) to 100 (no pain and perfect function). This tool demonstrated good test–retest reliability, face, content, and construct validity and high clinical change responsiveness [21].

### 2.6. The Hip Outcome Score (HOS)

The HOS is an established 31-item self-reported questionnaire used for evaluating activities, limitations in everyday life, and quality of life of patients with a hip disorder [22]. It comprises activity of daily life and sports activities subscales answered retrospectively (considering the past week). Scores range from 0 (worst function and lowest level of activity) to 100 (best function and level of activity).

### 2.7. Psychometric Properties’ Analysis

The SPSS software version 27 (IBM SPSS Statistics, Chicago, IL, USA) was used for all of the statistical analyses. Statistical significance was set at 5% (*p* < 0.05).

Internal consistency

Cronbach’s alpha was used to analyze the internal consistency of the Spanish version of the IHOT-12. A Cronbach’s alpha value ≥ 0.70 is considered acceptable [23]. Structural validity was assessed through an EFA to identify the optimal factor structure. Thus, to identify whether the Pearson correlation matrix was factorizable, the Barlett’s test and the Kaiser–Meyer–Olkin (KMO) test were used [24]. According to the statistical recommendations, the optimal number of factors was established based on the parallel analysis method [25,26]. A principal axis factoring method with oblique rotation was conducted as a factor extraction method. For the item inclusion in each factor, a factor loading > 0.4 was considered necessary.

### 2.8. Convergent Validity

The convergent validity of the Spanish version of the IHOT-12 was evaluated through its relationship with the IHOT-33 and with the HOS. These correlations were evaluated using Spearman correlation coefficients according to the cutoff values established by Domholdt: very weak (≤0.25), weak (0.26–0.49), moderate (0.50–0.69), strong (0.70–0.89), and very strong (≥0.90) [27].

### 2.9. Test–Retest Reliability

Test–retest reliability of the IHOT-12 was examined using the intraclass correlation coefficient and a value of ≥0.70 was considered acceptable [28]. The precision was measured by the standard error of measurement (SEM) [29], which was calculated as follows: $\mathrm{SD}\times \sqrt{1-\mathrm{ICC}}$. In addition, the minimal detectable change (MDC) [30] was calculated as $\mathrm{SEM}\times \sqrt{2}\times 1.96$.

### 2.10. Floor and Ceiling Effects

Floor and ceiling effects may affect the validity and reliability of the Spanish version of the IHOT-12. The percentage of patients who obtained the minimum and the maximum score on the questionnaire was calculated. The floor/ceiling effect was assumed to be present if at least 15% of patients achieved the minimum/maximum score, respectively [31].

## 3. Results

One hundred and fifty-three patients were finally analyzed in the study, of whom 64.7% were male (99 male and 54 female). Age ranged from 20 to 59 years, presenting a symptom duration between 3 and 18 months. Anthropometric and clinical characteristics are provided in Table 1.

**Internal consistency**

**Figure 1:**
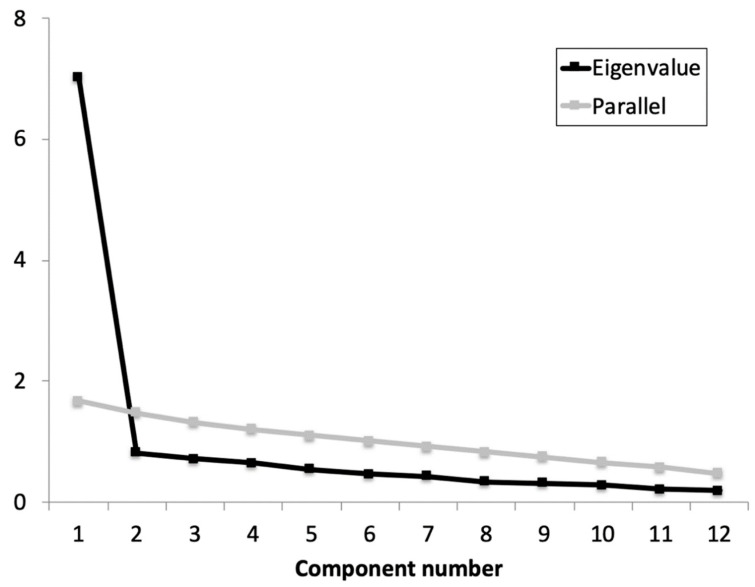
Parallel analysis and eigenvalue for each component of the iHOT-12Sv.

Previously to the EFA, the Cronbach’s α coefficient was calculated for the whole scale and the adjusted item-total correlations. Based on the results obtained (α = 0.92 (0.91 to 0.96); average item-total correlations ≥ 0.641), no items were removed as all contributed substantially to the scale. The KMO showed an acceptable data suite for factor analysis (KMO = 0.934) and Barlett’s test of sphericity refused the identity matrix null hypothesis: χ^2^ (66) = 639.22, *p* < 0.001. The parallel analysis suggested retaining one factor that explained 57.9% of the variance and an eigenvalue of 7.0 (Figure 1). All items showed an optimal factor loading (≥0.659).

**Convergent Validity**

The correlations of the iHOT-12Sv with the other measures to determine its convergent validity are shown in Table 2. The iHOT-12Sv total score showed a strong correlation with the HOS total score, as well as a very strong correlation with the IHOT-33. Thus, patients with greater iHOT-12Sv scores presented a greater score in both the HOS and IHOT-33 questionnaires.

**Test–retest reliability**

To assess the test–retest reliability of the IHOT-12, a total of 153 patients (gender male: 64.7%; age: 39.72 ± 7.90 years; body mass index: 23.86 ± 1.54 Kg/m²) re-filled the questionnaire after 14 days. According to the intraclass correlation coefficient (ICC), the stability over time of the scale was excellent, with the MDC_95_ being established at 17.22. The descriptive statistics, as well as the results of the test–retest reliability and responsiveness analysis, for the IHOT-12 Spanish version are shown in Table 3.

**Table 3:** Test–retest reliability measures of the Spanish version of the iHOT-12Sv.

	Mean ± SD		Mean Difference (95%CI)	ICC (95% CI)	SEM	MDC_95_
	Test 1	Test 2				
**iHOT-12Sv**	**47.61 ± 16.35**	**45.99 ± 15.52**	**1.62 (−0.48; 3.57)**	0.93 (0.86; 0.94)	6.21	17.22

ICC, intraclass correlation coefficient; iHOT-12Sv, Spanish version of the International Hip Outcome Tool-12; MDC, minimal detectable change; SEM, standard error of the mean; CI, confidende interval.

**Floor and Ceiling Effects**

The final Spanish version of IHOT-12 consisted of 12 items formulated in a direct/positive manner and rated by placing a mark on a 100 mm visual analogue scale (0 indicates “significantly impaired” and 100 indicates “no problem at all”). Higher scores indicate higher levels of physical function. No floor or ceiling effects were found for the average score on the IHOT-12. Specifically, only two patients obtained the lowest possible score (1.3%), while the highest possible score was obtained by only six patients (3.9%).

## 4. Discussion

This study aimed to determine the validity and reliability of the Spanish version of the iHOT-12 (iHOT-Sv) to assess the physical function and health-related quality of life in a population of young patients with femoroacetabular impingement with or without labral tear. Based on our results, the iHOT-12-Sv could be successfully recommended in physically active and young Spanish-speaking patients with hip conditions.

The internal consistency of the iHOT-Sv was excellent, demonstrating that all items contributed to the scale. These results are reinforced by previous validation studies [8,10,12], obtaining in most of them a Cronbach’s alpha >0.90 (e.g., the French version obtained a Cronbach’s alpha = 0.86) [11], but without obtaining a value high enough to reflect the presence of redundant items. Internal consistency is an important aspect to determine the homogeneity among the items on a test for verifying whether the items are consistent with each other measuring the same thing [32]. In addition, according to EFA findings, the final version of the iHOT-Sv consisted of 12 items englobed into one of the original factors, resulting in a reasonably good model and obtaining an optimal factor loading (≥0.641). Hence, the iHOT-12-Sv presented a unifactorial model, in line with the originally created version of the scale and with previous validations to other languages [7,8,9,10,11,12]. On the other hand, in line with previous validations of the iHOT-12 scale [8,12], our results showed no floor or ceiling effects. Therefore, this version shows an adequate discriminant capacity and could be used for measuring changes in physical function and quality of life in young and active patients with hip pathology to the detriment of other measurement instruments such as the Harris Hip Score whose validity in this type of population has been questioned [33].

Convergent validity was evaluated with the HOS and the iHOT-33 as they are applicable for assessing outcomes of treatment interventions in this patient population and were previously validated in the Spanish language [13,31]. The iHOT-12-Sv demonstrated strong correlations with the HOS and a very strong correlation with the iHOT-33. Thus, both subscales of the HOS analyzed independently showed strong correlations. Therefore, this iHOT-12-Sv could be considered a valid, short, and simple alternative to the full version for its use in prospective clinical research.

The iHOT-12Sv demonstrated excellent test–retest reliability. Our reliability estimates (ICC = 0.93) were comparable to the French (0.84), Swedish (0.88), English (0.89), Turkish (0.92), Dutch (0.93), and German (0.94) versions of the iHOT-12 [7,8,10,11,12]. No systematic bias was found between the first and the second attempts. In addition, the MDC was similar to those obtained in previous validations of the scale [8,10,11,12]. Specifically, according to our results, a change in the iHOT-12-Sv score equal to or greater than 17.21 points would indicate that it is a real change and not due to measurement error. It is important to mention that all of the items of the iHOT-12 are included in the iHOT-33 [4]. Thus, although they are not totally comparable, it could be considered that the Spanish version of 12 items is somewhat less sensitive to change than the extended version of 33 items, as the latter presents an MDC of 12.5 points. Logically, this difference could be attributed to the greater precision obtained by increasing the number of items. However, we consider that the slight increase in sensitivity to change does not compensate for the greater time invested in completing the extended version. Based on the above, we consider that the iHOT-33 could be a more interesting scale to apply in the research setting, whereas the application of the iHOT-12 in the clinical setting could be more relevant and more widely accepted.

### Limitations

Some limitations of this study must be acknowledged. Firstly, this study used a convenience sample, which could affect the extrapolation of the results to other hip disorders. Another limitation is that the Spanish language used in Spain differs from the Spanish used in other Spanish-speaking countries, so it is unknown whether the metric properties of the scale would be maintained when applied in other Spanish-speaking countries owing to linguistic and cultural differences. Finally, the degree of improvement or change perceived by the patients (subjective assessment) was not assessed, so the minimum clinically important difference could not be determined.

## 5. Conclusions

The Spanish version of the iHOT-12 (the i-HOT12Sv) is a valid, reliable, and fast tool for evaluating the hip function in physically active patients with labral tear injury and/or FAI Cam or Pincer type. Despite that the i-HOT12Sv could be more appropriate for clinical purposes, the extended version is recommended for research aiming to assess changes in hip function.

## Figures and Tables

**Table 1:** Anthropometric and clinical characteristics.

Anthropometric Characteristics	Mean ± SD or N (%)
**Age (years)**	39 ± 7.9
**Gender (male)**	99 (64.7%)
**Height (cm)**	177 ± 11
**Weight (Kg)**	75.6 ± 9.7
**Clinical characteristics**	
**Duration of symptoms (months)**	7.46 ± 3.29
**Visual Analogue Scale (100-mm)**	58.47 ± 18.48
**Hip Outcome Score (HOS)**	63.48 ± 15.51
**International Hip Outcome Tool**	
12 items (iHOT-12S)	47.59 ± 19.12
33 items (iHOT-33)	51.03 ± 18.19
**Diagnosis**	
FAI Cam type	47 (30.7)
FAI Cam type + labral tear	27 (17.6)
FAI Pincer type	30 (19.6)
FAI Pincer type + labral tear	19 (12.4)
Labral tear	30 (19.6)

**Table 2:** Convergent validity of the Spanish version of the iHOT-12Sv.

	Spanish Version of the International Hip Outcome Tool-12 (iHOT-12Sv)	
	Spearman’s Rho	*p*-Value
**Hip Outcome Score (HOS)**		
Activities of Daily Life subscale	0.772	**<0.001**
Sport subscale	0.729	**<0.001**
TOTAL score	0.836	**<0.001**
**International Hip Outcome Tool 33 (IHOT-33)**	0.938	**<0.001**

## Data Availability

All data derived from this study are presented in the text.

## Supplementary Materials

The following supporting information can be downloaded at: https://www.mdpi.com/article/10.3390/jcm11216232/s1, File S1: Herramienta Internacional DE Resultados DE Cadera (iHOT-12).
