# Supplementary material for: Validity and Test–Retest Reliability of the Spanish Version of the International Hip Outcome Tool (iHOT-12Sv)
Source: J Clin Med. 2022 Oct 22;11(21):6232. doi: 10.3390/jcm11216232 (PMC9655660; doi:10.3390/jcm11216232)
Supplement: Supplementary file 1 [file jcm-11-06232-s001.zip › jcm-1976599-supplementary.pdf]

HERRAMIENTA INTERNACIONAL DE RESULTADOS DE CADERA (iHOT-12Sv)

Datos del Paciente:	¿A qué cadera se refiere esta encuesta?
Nombre:<br><br>Fecha de nacimiento:	Si le hemos indicado que responda la encuesta respecto a una cadera en particular, marque esa. Si no le hemos dicho nada marque la que más le moleste.<br><br>☐ Derecha ☐ Izquierda

Cuestionario de Calidad de vida para personas jóvenes y activas con problemas de cadera

INSTRUCCIONES

Estas preguntas se refieren a los problemas que pueda estar usted experimentando en su cadera, a la manera que estos problemas afectan a su vida y a las emociones que le desencadenan estos problemas.

Por favor, indique la gravedad de los síntomas poniendo una marca sobre la línea que hay bajo cada pregunta.

- Si usted pone la marcha en el extremo izquierdo, esto quiere decir que usted considera que está muy afectado. Por ejemplo:

Afectada de forma significativa / ________________________________ Sin ningún problema

- Si usted pone la marcha en el extremo derecho, significa que usted no cree tener ningún problema relacionado con su cadera. Por ejemplo:

Afectada de forma significativa ________________________________ / Sin ningún problema

- Si pone la marca en el centro de la línea esto indicará que usted se encuentra moderadamente incapacitado, en otras palabras, entre los extremos de “afectado de forma significativa” y “sin ningún problema “. Es importante poner la marca más hacia los extremos de la línea si las descripciones más extremas reflejan con más precisión su situación.

Por favor, intente que sus respuestas reflejen su situación habitual durante **el último mes**.

Consejo Si usted no realiza una actividad sobre la que se le pregunta, imagine como afectaría a su cadera si tuviera que intentarlo.

P1 En conjunto ¿Cuánto dolor tiene en su cadera o ingle?

Dolor intenso ______________________________ Ningún dolor

P2 ¿Tiene dificultades para levantarse y acostarse en el suelo?

Dificultad extrema ______________________________ Ninguna dificultad

---

P3 ¿Cuánta dificultad tiene usted para caminar largas distancias?

Dificultad extrema ______________________________ Ninguna dificultad

---

P4 ¿Tiene molestias debido a los chasquidos, crujidos o bloqueos de su cadera?

Molestias intensas ______________________________ Ninguna molestia

---

P5 ¿Tiene dificultad para empujar, tirar, levantar o transportar objetos pesados?

Dificultad extrema ______________________________ Ninguna dificultad

---

P6 ¿Cuánto le preocupa no poder giros rápidos mientras hace deporte o actividades de ocio?

Muy preocupado ______________________________ No me preocupa

P7 ¿Cuánto le duele la cadera después de hacer actividad física?

Dolor intenso ______________________________ Ningún dolor

P8 ¿Le preocupa no poder coger o llevar niños por su dolor de cadera?

Dolor intenso ______________________________ Ningún dolor

P9 ¿Tiene dificultades durante la actividad sexual debido a su cadera?

☐ Esto no me parece relevante

Dificultad extrema ____ Ninguna dificultad

P10 ¿Cuánto tiempo es consciente de la discapacidad que le provoca su cadera?

Constantemente ____ Nunca

P11 ¿Cuánto le preocupa su capacidad para mantener el nivel de forma física que desea?

Muy preocupado ____ No me preocupa

P12 ¿Cuánta distracción le provoca su problema cadera?

Mucha distracción ____ Ninguna distracción
